# Non-Genetic Factors in Schizophrenia

**DOI:** 10.1007/s11920-019-1091-3

**Published:** 2019-09-14

**Authors:** Simona A. Stilo, Robin M. Murray

**Affiliations:** 1 Department of Psychosis Studies, Institute of Psychiatry, Psychology & Neuroscience, King’s College London, De Crespigny Park, London, SE5 8AF UK; 2 National Institute for Health Research (NIHR) Mental Health Biomedical Research Centre at South London and Maudsley NHS Foundation Trust and King’s College London, London, UK

**Keywords:** Schizophrenia, Psychosis, Risk factors, Environment, Gene–environment interaction, Childhood trauma, Cannabis

## Abstract

**Purpose of Review:**

We review recent developments on risk factors in schizophrenia.

**Recent Findings:**

The way we think about schizophrenia today is profoundly different from the way this illness was seen in the twentieth century. We now know that the etiology of schizophrenia is multifactorial and reflects an interaction between genetic vulnerability and environmental contributors. Environmental risk factors such as pregnancy and birth complications, childhood trauma, migration, social isolation, urbanicity, and substance abuse, alone and in combination, acting at a number of levels over time, influence the individual’s likelihood to develop the disorder.

**Summary:**

Environmental risk factors together with the identification of a polygenic risk score for schizophrenia, research on gene–environment interaction and environment–environment interaction have hugely increased our knowledge of the disorder.

## Introduction

Schizophrenia has a well-established genetic component, which can now be estimated using the polygenic risk score for schizophrenia [1, 2••]. In the ground-breaking meta-analysis of genome-wide association study (GWAS) of schizophrenia, 108 schizophrenia-associated loci were identified [2••]. The loci implicated include genes involved in dopamine synthesis, calcium channel regulation, immunity, and glutamate neuroreceptors. However, this and subsequent GWAS studies explain only a minority of the variance in the liability for schizophrenia in the general population. This reflects the fact that a significant proportion of the liability may be due to gene–environment interactions [3] or to epigenetic mechanisms reflecting the effect of environmental factors. Indeed, growing evidence shows that non-genetic risk factors not only contribute to the illness but also suggest ways in which we may find potential subgroups of subjects at higher risk and therefore influence clinical management.

### Pregnancy and Birth Complications

Obstetric complications are well-documented as a risk factor for schizophrenia [4•,^5^–^7^]; increased susceptibility has been associated with emergency cesarean section, bleeding during pregnancy, preeclampsia [6], and low birth weight [8–10]. Use of forceps and low birth weight predict earlier age of onset of psychosis [11].

Some epidemiologic studies have also suggested that exposure to viruses and other infectious agents such as influenza, toxoplasmosis, and herpes simplex virus type 2, contracted during pregnancy [12, 13] and around the time of conception [14], are associated with a later risk of psychotic disorders. However, these results have not always been replicated. For example, a recent meta-analysis by Selten et al. has shown insufficient evidence to prove an association between second-trimester influenza exposure and psychotic outcome in the offspring [15••].

Four main pathogenic mechanisms have been suggested as involved: fetal malnutrition, prematurity, hypoxic-ischemic events, and maternal infections during pregnancy or delivery [4•, 16–20]. Moreover, elevated markers of inflammation, including maternal C-reactive protein [21] and interleukin-8 [22] have been found in mothers of patients with schizophrenia. Late winter or spring birth has often been reported as risk factor for schizophrenia [8, 23–25]. However, it has a very small effect [26]; whether it is secondary to maternal infection or nutritional deficiency remains unclear.

### Advanced Parental Age

Increased paternal age, from age higher than 34 and upwards [27, 28], has been associated with schizophrenia [29–31]. An attractive theory suggests that age-associated increase in sporadic de novo mutations in male germ cells may play a role [32–34]. However, this was discounted by a study from Denmark that suggests that late marriage and reproduction may be due to personality attributes of fathers [35].

A less consistent pattern of findings has emerged regarding maternal age at birth and risk of schizophrenia in offspring. In one study, age younger than 19 and age older than 40 years [36] appeared to increase the risk. However, in another cohort study, the risk appeared decreased in offspring of mothers older than 30 years [37]. Lopez-Castroman et al. (2010) found a significant linear association increase only with advancing maternal age [38].

### Trauma and Social Adversities

Trauma and social adversities in different forms, either during childhood or adulthood, have been extensively investigated as potential risk factors for schizophrenia. Varese and colleagues, in a meta-analysis of case-control, prospective, and cross-sectional cohort studies, reported strong evidence that childhood adversity (defined as sexual abuse, physical abuse, emotional/psychological abuse, neglect, parental death, and bullying) was associated with increased risk for psychosis in adulthood (overall OR = 2.78) [39•]. There is an association between permanent separation from, or death of, one or both parents and psychosis [40–43], victimization and bullying and psychosis [44–46]. A robust link between childhood trauma and schizophrenic symptoms has been found [47–49] with childhood trauma being associated with the most severe forms of positive symptomatology in adulthood, particularly hallucinations [49–51], and affective symptoms [52]. Life events more proximal to the onset of illness, defined as situations that bring about positive or negative changes in personal circumstances and/or involve an element of threat, have been investigated [53–55]. The most recent review and meta-analysis of the relationship between life events and psychosis has suggested around a threefold increased odds of life events in the period prior to psychosis onset, with the time period under consideration ranging between 3 months and 3.6 years [55].

### Social Class and Isolation

Some reports link social inequality at birth with schizophrenia. Socioeconomic status (usually measured by paternal occupation) has been reported to be associated with an increased risk of psychosis [56–60]. However, while some findings are positive, there are a number of conflicting studies showing no association between psychosis and low social class at birth [41] or even a link with high social class [61, 62].

Markers of isolation/disadvantage, alone and cumulative, are also associated with psychosis [42, 43, 63]. First-episode psychosis patients are more likely to live alone; be single or unemployed; live in a rented accommodation, in overcrowded conditions; and receive an income below official poverty, not only at first contact with psychiatric services but up to 5 years prior to the onset of psychosis, with around a twofold increased odds [43]. The World Health Organization (WHO) studies have reported that despite the better access to biomedical treatment, higher rates of chronic disability and dependency in schizophrenia occur in high- than low-income countries and suggest that something essential to recovery is missing in the social fabric [64].

### Migration

Meta-analytic reviews show that migrant groups are at increased risk of schizophrenia and other psychotic disorders [65, 66•]. These findings have been consistently replicated in a number of high-income countries: the UK [67], the Netherlands [68], Germany [69], Denmark [70], France [71], Italy [72], and to a lesser extent Canada [73] with some evidence that the risk of schizophrenia and other non-affective psychotic disorders is especially high among refugees compared with non-refugee migrants [74]. Interestingly, the risk appears to persist into the second and third generations [66•, 75].

The level of risk appears to vary by country of origin. A recent meta-analysis of schizophrenia incidence in the UK reported almost a five times greater risk of schizophrenia among people of black Caribbean origin compared with reference UK population (usually white) [76]. Numerous hypotheses have been tested. Higher incidence rates in the country of origin, selective migration, or misdiagnosis of mood disorders do not seem to explain the phenomena [77–81]. However, social adversity exposures at all stages of the migration process (before, during, and after) [82], low ethnic density [83], social isolation [84], discrimination [68], and lack of access to private accommodation and economic opportunities [85] have all been suggested as contributing, so has vitamin D deficiency [86, 87] but as yet little direct evidence for the latter has been found.

### Urbanicity

Growing up and/or living in an urban environment has frequently been associated with an increased risk of schizophrenia or psychosis in general [88–91]. A meta-analysis, including a total of 47,087 cases with psychosis, shows a pooled OR for psychosis in urban environment compared with the rural environment of 2.39 (95% CI 1.62–3.51) [92•]. Changing residence in childhood from rural to urban environment doubles the risk of developing schizophrenia [93, 94], and the more years a child spends in an urban area, the greater the risk becomes [95]. Many explanations have been proposed such as greater exposure to prenatal influenza [96], maternal obstetrical complications [97, 98], toxoplasma gondii infection [99], cannabis use [100], social deprivation, income inequality, and social fragmentation [101, 102] but none of them has been verified. Furthermore, while the largest multicentric study of first-episode psychosis patients to date (EU-GEI study) confirmed higher incidence in Northern European cities including London, Amsterdam, and Paris, the increased density effect is not so clear in Southern European settings [103•].

Interestingly, a recent study conducted in Denmark, has pointed out for the first time the protective role of living in, or near to, a green area, showing a dose-response association between the magnitude of greenspace during childhood and the risk of later development of schizophrenia [104•].

### Cannabis and Other Substance Use

Substance use is highly prevalent in psychotic patients [105–107]. There is good evidence that psychostimulants (such as amphetamines and cocaine) can induce psychosis [108]. There also have been a few suggestions that alcohol misuse and psychosis might be associated [109, 110], and recently, a meta-analysis raised the question of whether tobacco use could be a risk factor for psychosis [111]. However, much greater evidence points to an important aetiological role for cannabis use. Prospective epidemiological studies consistently report an association between cannabis use and schizophrenia [112–114] with an estimated two- to threefold increased risk [114, 115]. A dose–response relationship between extent of use and risk of psychosis has been shown in a meta-analysis [116]. The association is stronger in those individuals who used cannabis earlier [113], and who used high potency tetrahydrocannabinol (THC) cannabis or/and more frequently [112, 117, 118]. Indeed, the EU-GEI study has found that if high-potency cannabis was no longer available, around 12% of first-episode psychosis cases across 11 Europe-wide sites could be prevented, rising to 30% in London and 50% in Amsterdam [119•]. The age at which cannabis use begins appears to correlate with the age at onset of psychosis [118, 120, 121] while persistent cannabis use after a first episode is associated with poorer prognosis [122, 123], higher relapse rates, longer hospitalizations, and severe positive symptoms [124].

### Cognitive Impairments and Brain Structural Abnormalities

Although schizophrenia usually manifests in adolescence and early adult life, numerous reports suggest that many patients with schizophrenia have a history of delayed developmental milestones in the first year of life [125], lower IQ in childhood [126–128], hearing impairment [129], emotional problems, and interpersonal difficulties early in life [130, 131]. Those people who develop psychosis following heavy cannabis use show less evidence of such neurodevelopmental deviance. In particular, they have higher premorbid IQ and better social functioning in childhood than psychotic patients who do not use cannabis [132]. Perhaps being smarter and more sociable enables them to find cannabis dealers and obtain the money for the drug!

### Gene–Environment Interaction

**Fig. 1:**
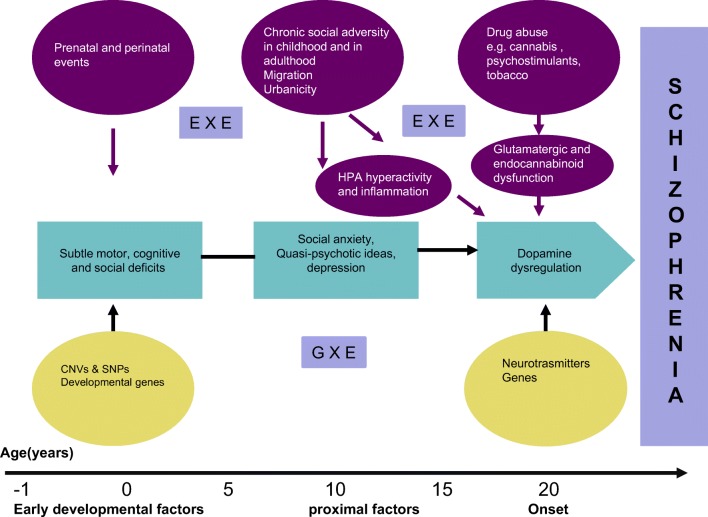
Developmental cascade towards schizophrenia. CNV, copy number variations; SNPs, single nucleotide polymorphisms; ExE, environment–environment interaction; GxE, gene–environment interaction

It is becoming increasingly clear that none of the risk factors discussed above, by itself, is either necessary or sufficient for the development of schizophrenia. Most of show a modest effect (twofold increase in risk) and none seem specific for schizophrenia. Different factors operating at various levels contribute to onset and progression of the disorder. The developmental cascade towards schizophrenia [133] should now include gene–environment interaction (GXE), and environment–environment interaction (EXE) (Fig. 1).

Interest has turned to the possibility of gene × environmental interactions. Preliminary reports suggested interactions between functional polymorphisms in the catechol-O-methyltransferase gene [134], or the *DRD2* genotype (OMIM 126450) [135], and the AKT1 genotype (C/C rs2494732) [136, 137] and cannabis use, on risk of psychosis. However, all of these studies are relatively small and in need of replication. A few preliminary studies have examined interactions between the polygenic risk score for schizophrenia (e.g., Trotta et al. [138]; Ursini et al. [139]) but there are not sufficient studies yet to evaluate this adequately. The largest and most recently published study to date, analyzing the associations of polygenic risk score for schizophrenia and environmental exposures in 1699 patients and 1542 unrelated controls, shows an additive interaction between polygenic risk score and lifetime regular cannabis use and exposure to early life adversities (sexual abuse, emotional abuse, emotional neglect, and bullying), but not with the presence of other exposures such as hearing impairment, winter birth, physical abuse, or physical neglect [140••] confirming the need for future confirmatory studies.

### Cumulative Effect of Environmental Risk Factors

A few studies have now started examining the additive effect of multiple environmental factors on risk of psychosis as aggregate index of total number of risk factors or weighted sum. Cougnard et al. reported an additive interaction between exposure to three risk factors—cannabis use, childhood trauma, and urbanicity—and baseline psychotic experiences in predicting persistent psychotic symptoms three years later in the general population [141]. Stepniak et al. found that individuals who had been exposed to 4 or more environmental risk factors had a significantly lower age of onset than those exposed to 3 factors [142]. As a predictor tool, Padmanabhan et al., in a pilot study, explored the association of cumulative environmental risk (including nine risk factors) with conversion to psychosis in a family high-risk population [143].

### Neurochemical Mechanisms

Different systems: dopaminergic (DA), glutamatergic, neuroinflammation/immune, and more recently endocannabinoid (eCB), have all been investigated to understand the exact mechanism(s) by which some non-genetic risk factors can affect brain function. The predominant biological theory of schizophrenia highlights the role of the excess presynaptic synthesis of DA in the striatum in the onset of positive symptoms [144•, 145]. Consistent with this view, the diathesis–stress model suggests that the hypothalamus–pituitary–adrenal (HPA) axis may trigger a cascade of events resulting in neural circuit dysfunction, including alterations in DA signaling [146]. Epidemiological studies are consistent with a key role of stress/cortisol in the onset of psychosis. Higher levels of diurnal cortisol have been reported in patients compared with controls or patients on antipsychotic treatments for less than 2 weeks [147] and high baseline cortisol levels appear to facilitate transition to psychotic level symptoms in at-risk youths [148]. Mizrahi et al., investigating the DA release in response to a psychosocial stress challenge in psychosis-related disorders, found that the largest stress-induced changes in salivary cortisol was present in the schizophrenia group, followed by the clinical high-risk group, with an association between the percent change in the cortisol response and the stress-induced DA release in the associative striatum [149].

New data provide intriguing evidence of an association between migration [150], hearing impairment [151], childhood abuse [152], low parental care [153], and elevation in striatal dopamine synthesis. Acute administration of THC, the active ingredient of cannabis, has been reported to increase dopamine release [154]. However, paradoxically, chronic cannabis use [155] and also difficult premature birth [156] are associated with decreased striatal dopamine. Perhaps DA receptor sensitivity or dysregulation in response to stress may be one pathway through which the different exposures interact with genetic vulnerability to confer a higher risk of schizophrenia [144].

A compelling case is made for the role of glutamate/NMDA receptors in schizophrenia, originally suggested as a mechanism underlying the psychotogenic effects of PCP and ketamine [157]. There is now strong evidence in support of the hypothesis that hypofunction of NMDA receptors contributes to the symptoms of schizophrenia [158, 159]; some reports suggest that dopamine dysregulation in schizophrenia may be secondary to glutamatergic dysfunction in some cases at least [160, 161]. Furthermore, glutamatergic neurotransmission has been shown to mediate the effects of both acute and chronic stress [162].

Just as amphetamine-induced psychosis gave rise to the dopamine hypothesis and ketamine-induced psychosis to the glutamate hypotheses, so cannabis-induced psychosis has provoked interest in the endocannabinoid system [163]. Certainly, the endogenous cannabinoid system (eCB) is altered in schizophrenia. The CB1 receptor densities and anandamide levels have been reported abnormally in patients with schizophrenia [164, 165]; among other function, the eCB system appears to regulate the HPA axis [166–168]. It has been suggested that a dysregulation of this system (that could be induced for example by exogenous cannabis) can interact with neurotransmitter systems in such a way that an “endocannabinoid hypothesis” can be integrated into the neurobiological hypotheses of schizophrenia [164].

Another possible molecular mechanism underlying psychosis risk is neuroinflammation and abnormalities of the immune system [169, 170•]. A recent meta-analysis examining peripheral inflammatory markers shows that some markers such as interleukin 6 (IL-6), tumor necrosis factor α (TNFα), soluble IL-2 receptor (sIL-2R), and IL-1 receptor antagonist (IL-1RA) increase in acute episodes and tend to decrease after successful treatment [171]. The effects of childhood trauma on inflammation have been well-studied. Individuals exposed to childhood trauma have significantly elevated baseline peripheral inflammatory markers in adulthood [172, 173]. Another recently studied hypothesis implicates the immune system. Autoimmune diseases are reported to occur in 3.6% of patients with schizophrenia [174]. Mechanisms through which systemic immune activation affect risk of psychopathology include the effects of inflammation on concurrent brain function, the effects of early immune activation on brain development, the sensitization of immune brain cells to subsequent psychosocial stressors, and the cross-sensitization of the HPA axis response to subsequent psychosocial stressors [172]. It has been speculated that inflammation-mediated pathways may serve as a final common pathway for environmental risk factors such as early childhood adversity, adolescent cannabis use, and social exclusion [170]. However, hard evidence supporting this hypothesis remains elusive.

## Conclusions

Epidemiological studies have consistently shown a pattern of association between environmental risk factors and later onset of psychosis, which is suggestive of a causal relationship. However, there are a number of reasons why the association between environmental risk factors and psychotic outcomes may be overestimated or underestimated such as bias (where incorrect estimates are due to measurements or sample selection), chance, confounding (third explanation for the association), and reverse causation (where psychosis increases risk of an environmental exposure), which should be taken in consideration when causality is inferred. Studying gene–environment interaction and gene–environment correlation (rGE) (genetic effects on environment exposure) may clarify the position [175, 176].

Future research should also explore potential protective factors in groups who have a lower risk of psychotic disorders. A new field of research includes big data and predictive models, where traditional paper notes have been replaced with electronic patient records. In line with this direction, there have been initial successful applications of machine learning algorithms to diagnose psychosis [177]. As with diagnostic tools for cardiovascular risk, schizophrenia will in the near future probably require a combination of diagnostic approaches, including measures of genetic risk, environmental risk factors, and imaging.
